# Suicidal Feelings Interferes with Help-Seeking in Bullied Adolescents

**DOI:** 10.1371/journal.pone.0106031

**Published:** 2014-09-04

**Authors:** Yuko Kitagawa, Shinji Shimodera, Fumiharu Togo, Yuji Okazaki, Atsushi Nishida, Tsukasa Sasaki

**Affiliations:** 1 Department of Health Education, Graduate School of Education, University of Tokyo, Tokyo, Japan; 2 Department of Neuropsychiatry, Kochi Medical School, Kochi University, Kochi, Japan; 3 Tokyo Metropolitan Matsuzawa Hospital, Tokyo, Japan; 4 Department of Psychiatry and Behavioral Science, Tokyo Metropolitan Institute of Medical Science, Tokyo, Japan; Chiba University Graduate School of Medicine, Japan

## Abstract

**Purpose:**

Being bullied is associated with the manifestation of suicidal feelings, which sharply increase in middle(-late) adolescence. Whether or not bullied middle(-late) adolescents with suicidal feelings seek help is therefore a critical issue, given that help-seeking plays a key role in the prevention of suicide. The aim of the present study is to investigate the effects of bullying, suicidal feelings and the interaction between these two factors on help-seeking behavior in adolescents.

**Methods:**

Japanese middle(-late) adolescents (aged 15–18 years; n = 9484) were studied using self-report questionnaires. The rate of adolescents who actually sought help was examined for bullying status and suicidal feelings.

**Results:**

The rate of adolescents who sought help was significantly higher when they were bullied (*p*<0.001) and also when they had mild suicidal feelings (*p*<0.001), but not when they displayed serious suicidal feelings. In the case of adolescents who were bullied, however, having suicidal feelings significantly decreased the rate of help-seeking (OR = 0.47, *p*<0.05 and OR = 0.32, *p* = 0.002 for having mild and serious suicidal feelings, respectively). The decrease was remarkable when suicidal feelings were serious. Specifically, the decrease was significant in seeking help from peers and family members, who are the most frequent source of the help for adolescents, when they had serious suicidal feelings (OR = 0.21, *p*<0.01 and OR = 0.13, *p*<0.001, respectively).

**Conclusions:**

Suicidal feelings may interfere with help-seeking behavior, which could be critical in suicide prevention in bullied middle(-late) adolescents.

## Introduction

Suicide is a leading cause of death in adolescence. The rate of suicide increases dramatically during the teenage years [1]–[3]. Worldwide, the total number of suicides among people in early adolescence (aged 10–14 years) in 2004 was 11,000 (the tenth leading cause of death), but this jumped to 60,000 (the second leading cause of death) among people in middle(-late) adolescence (aged 15–19 years) [2]. In developed countries, suicide is the second leading cause of death in those aged 15–19 years, following traffic accidents as the first leading cause of death. [2] In 2011, in Japan, the rate of suicide (per 10,000 people) in early adolescents was 1.3, but this increased to 8.5 in middle(-late) adolescents [4]. Thus, prevention of suicide, especially in these adolescents, is one of the most significant issues in adolescent mental health [2], [3], [5], [6].

While suicide and suicidal behaviors are generally multifactorial [3], [5]–[7], bullying and help-seeking may play significant roles in these behaviors and their prevention in adolescents [3], [6], [8]–[13]. In adolescents, seeking help from non-health professionals, including peers, family members and teachers, and not only from professionals, is thought to play a major role in the prevention of suicide. Adolescents are more likely to seek help from non-professionals than professionals, and these non-professionals can serve as a pathway to professional services which can help prevent suicide [5], [12], [14].

Bullying is extremely prevalent in children and adolescents. A large-scale school-based epidemiological study observed that 30% of children in the U.S.(Grades 6–10^th^: 11–15 years)were involved in bullying, including as pure victims (those who are victimized and do not bully others; 11%), pure bullies (those who bully others and are not victimized themselves; 13%) and bully-victims (those who both bully and are victimized themselves; 6%) [15]. A school-based survey across 40 countries, including a number of European countries, Turkey, Israel and North American countries, found that 8.6–45.2% of children(11–15 years)were involved in bullying [16]. Several cross-sectional school-based studies observed that being bullied may be associated with the risk of suicidal behaviors in Finland, Korea and the U.S. [10], [17]–[20]. Longitudinal population-based cohort studies observed the association between victimization by bullying in childhood or adolescence and later mental disorders and suicidal behavior, including completed suicide, in the U.K. and Finland [21]–[24]. However, help-seeking behavior in bullied adolescents generally still remains to be studied. While some school-based studies in the U.K. have reported that bullied adolescents may be hesitant to consult others [25]–[27], studies in the U.K., Norway and the U.S. have observed that they may seek more help when bullied [28]–[30].

The relation between suicidal feelings and help-seeking is another important issue, which remains to be further studied. A U.S. epidemiological study found that two to three times more adolescents with suicidal feelings sought help, including formal help-seeking from health care professionals, than those without these feelings [31]. Australian and U.S. studies that investigated the intention to seek help, not actual behavior, however, observed a decreased intention in most adolescents with suicidal feelings [32], [33].

The present study therefore investigated the effects of bullying, suicidal feelings and their interaction on help-seeking behaviors in middle(-late) adolescents (aged 15–18 years). We hypothesized that suicidal feelings interfere with help-seeking in adolescents who are victims of bullying. The rate of adolescents who actually sought help was studied by bullying status and suicidal feelings using a large school-based sample. Help-seeking behaviors directed at different sources, including peers and family members (or informal help-seeking), not only health-care professionals (or formal help-seeking), were examined, given the fact that, as mentioned above, help from such sources is thought to play a role in reducing the risk of suicide [12], [14].

## Method

### Subjects

A cross-sectional survey of psychopathologies was conducted between 2008 and 2009 in Kochi prefecture (population: 790,000), Japan. Data were collected from students at 28 senior high schools (10^th^–12^th^ grades, aged 15–18 years). Out of 9,991 students at 28 senior high schools, 256 students (2.6%) were absent on the days of the survey, and 251 students (2.5%) did not agree to participate in this study. Thus, a total of 9,484 students (95.3% of 9,991, 4546 males and 4938 females, age: 16.6±0.93 years (mean ±s.d.), range  = 15–18 years) answered the questionnaire.

For the practical procedure of the study, the principal investigators approached the school principals and asked them to co-operate with the survey, and the principals then consulted with teachers and parents. After we had obtained written informed consent from the parents, the teachers handed a self-report questionnaire and envelope to each student. When they were distributed, the teachers explained to the students that: 1) participation in the study was anonymous and voluntary, and 2) strict confidentiality would be maintained. The students were requested to seal the completed questionnaire in the envelope provided. Research staff collected the sealed questionnaires at each school.

The study was planned and conducted in accordance with the Ethical Guidelines for Epidemiological Research of Japan, and approved by the ethics committees of the Tokyo Metropolitan Institute of Psychiatry, the Mie University School of Medicine, and the Kochi Medical School, Kochi University.

### Measures

The questionnaire included items on: 1) bullying and victimization, 2) suicidal feelings, 3) actual help-seeking behaviors, and 4) other variables including demographic characteristics such as sex and age. An expert in child and adolescent psychology and three teachers from the participating schools, including a Japanese language teacher, examined the questions for age-appropriateness and reading difficulty.

#### Bullying status

Experiences of being bullied or bullying others were assessed using the following questions: 1) “Have you been bullied within the past year?,” and 2) “Have you bullied others within the past year?” with a choice of two responses, “Yes” or “No.” Based on the responses, we classified the subjects into the following four groups: 1) those who did not bully and were not victimized (uninvolved); 2) those who bullied others and were not victimized themselves (pure bullies); 3) those who were victimized and did not bully others (pure victims); and 4) those who both bullied others and were victimized themselves (bully-victims).

#### Suicidal feelings

Suicidal feelings were measured using the following question: “Do you currently have thoughts that your life is no longer worth living?” [34] The participants selected one of four possible responses: “No”, “Probably not”, “Possibly yes”, or “Yes.” Based on the responses, we classified the subjects into the following three groups: 1) responses of “No” and “Probably not” were regarded as the absence of suicidal feelings (none), 2) responses of “Possibly yes” were regarded as the presence of mild suicidal feelings (mild), and 3) responses of “Yes” were regarded as the presence of serious suicidal feelings (serious).

#### Help-seeking behavior and resources for help

Actual help-seeking behaviors regarding psychological stresses were measured using the following question: “Have you recently consulted anyone to discuss your psychological stress or problems?” Participants selected one of two possible responses: 1) “No, I am not consulting anybody”, or 2) “Yes, I am currently consulting (or seeking help from) somebody about my stress or psychological problems.” The former was defined as “not seeking help”, and the latter as “seeking help”. If they selected 2) “yes”, they were asked to specify whom they were consulting or seeking help from. This question was answered by selecting one or more of the following: peers, family members, teachers, school nurses, psychologists, doctors or others.

### Statistical analysis

Associations between bullying status and suicidal feelings and between bullying status and the rate of adolescents who actually sought help were analyzed using the Kruskal-Wallis test, with post hoc comparison using the Bonferroni test.

The association between suicidal feelings and the rate of adolescents who actually sought help was examined by bullying status using logistic regression. The odds ratio for help-seeking in adolescents with mild or serious suicidal feelings was compared to those without suicidal feelings according to each bullying status. Sex and age were controlled for in the analysis. All statistical analyses were conducted using the Statistical Package for Social Science (SPSS), version 21.0 for Macintosh (IBM Inc., New York, U.S.).

## Results

### Frequencies of suicide feelings and bullying

The frequencies of suicidal feelings and bullying and their relations in the middle(-late) adolescents are summarized in Table 1. The frequencies of adolescents with mild and serious suicidal feelings were 815 and 434 out of 9,431 (8.6% and 4.6%) in the adolescents. Among these, the frequencies of pure bullies, pure victims and bully-victims were 4.0%, 3.1% and 1.1%.

**Table 1:** Frequencies of suicidal feelings by bullying status in middle(-late) adolescents.

		Suicidal feelings			
Adolescents	(n = 9431)	Mild	(n = 815)	Serious	(n = 434)
	n	n	(%)	N	(%)
Bullying status^b^					
Uninvolved	8657	706	(8.2)	324	(3.7)
Pure bullies^d^	372	42	(11.3)	42	(11.3)
Pure victims^c^	295	50	(16.9)	48	(16.3)
Bully-victims^c^	107	17	(15.9)	20	(18.7)

*Note*. Suicidal feelings are significantly associated with bullying status in middle(-late) adolescents (^b^ *p*<0.001, Kruskal-Wallis test). Severity of suicidal feelings is significantly higher in those bullied (pure victims and bully-victims) than in the uninvolved and pure bullies (^c^ *p*<0.001, Bonferroni post-hoc test), and the severity in pure bullies is significantly higher than in the uninvolved (^d^ *p*<0.001, Bonferroni post-hoc test) in middle(-late) adolescents. (The total number of subjects is less than the number of subjects analyzed, due to missing data for suicidal feelings or bullying status.)

Suicidal feelings were significantly associated with bullying status in the adolescents (*χ* ^2^ = 260.30, *d.f.* = 3, *p*<0.001, Kruskal-Wallis test). Suicidal feelings were significantly higher among those who were bullied (pure victims and bully-victims) than among the uninvolved and pure bullies (*p*<0.001 in Bonferroni post-hoc test), and were significantly higher among pure bullies than among the uninvolved adolescents (*p*<0.001 in Bonferroni post-hoc test).

### Frequencies of help-seeking by bullying status

As summarized in Table 2, the rate of adolescents who sought help was 37.6%. The Severity of suicidal feelings was significantly associated with the bullying status (*χ* ^2^ = 38.96, *d.f.* = 3, *p*<0.001, Kruskal-Wallis test). The frequency was significantly higher among pure victims (54.4%) than the uninvolved (36.8%) and pure bullies (41.2%) (*p*<0.001, with Bonferroni test).

**Table 2:** The number of the adolescents who sought help for psychological distress according to bullying status^a^ and sources of help.

	Total of subjects	(n = 8407)	Uninvolved	(n = 7734)	Pure bullies	(n = 318)	Pure victims	(n = 261)	Bully-victims	(n = 94)
	3161	37.6%	2844	36.8%	131	41.2%	142	54.4%	44	46.8%
Sources of help*										
**Informal help-seeking**	3003		2715		148		144		48	
Peers	2771		2492		127		115		37	
Family members	1192		1064		43		66		19	
Teachers	134		102		10		15		7	
**Formal help-seeking**	222		169		16		25		12	
(Health care professionals)										

*Note*. The number of students who sought help, divided according to the total number of adolescents, is shown by bullying status. The frequencies of the subjects who sought help are significantly associated with bullying status in the adolescents (^a^p<0.001, Kruskal-Wallis test). The frequency is significantly higher among pure victims than among the uninvolved and pure bullies (p<0.001, with Bonferroni test). *Multiple answers.

### Interactive effects of suicidal feelings and bullying status on help-seeking

**Figure 1:**
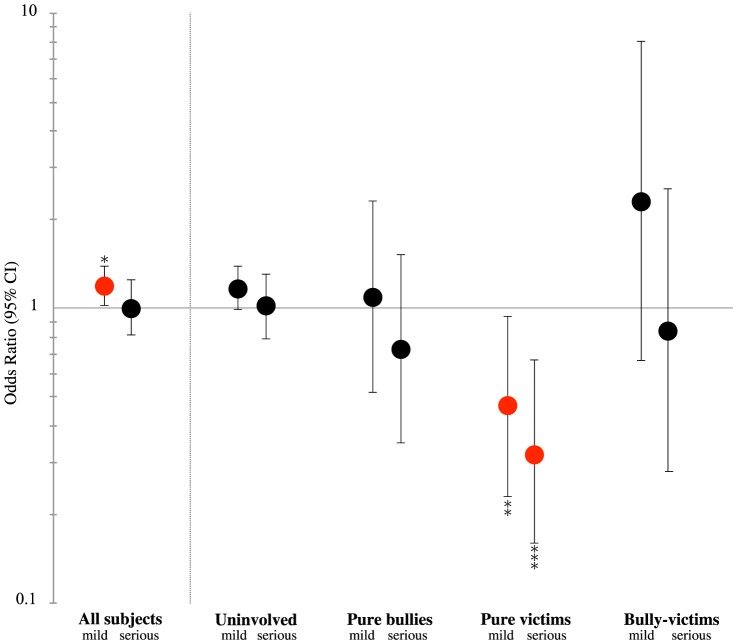
Interactive effects of suicidal feelings and bullying status on help-seeking for psychological distress in middle(-late) adolescents. *Note*. Odds ratio for seeking help (adjusted for gender and age); 95% CI = 95% confidence interval; mild  =  having mild suicidal feelings; serious  =  having serious suicidal feelings. Reference  =  having no suicidal feelings. In each section, subjects with missing data were excluded from the statistical analyses. *: *p*<0.05; **: *p*<0.01; ***: *p*<0.001.

**Figure 2:**
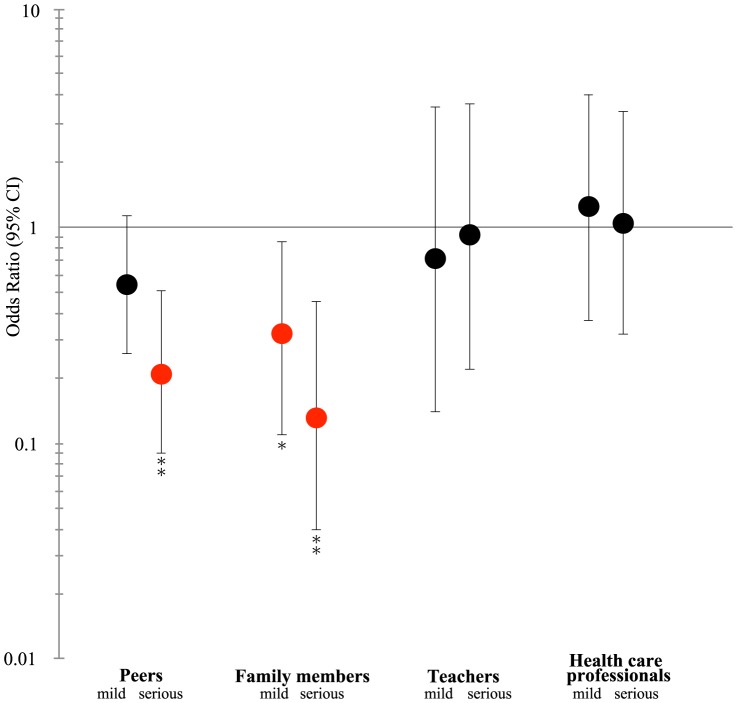
Effects of suicidal feelings on help-seeking for psychological distress by source of the help, in pure victims. Note. Odds ratio for seeking help from the source (adjusted for gender and age); 95% CI = 95% confidence interval; mild  =  having mild suicidal feelings; serious  =  having serious suicidal feelings. Reference  =  having no suicidal feelings. *: p<0.05; **: p<0.01.

The interactive effects of suicidal feelings and bullying status on help-seeking in the adolescents are summarized in Figure 1. In the total group of subjects, the rate of adolescents who sought help was significantly higher when they had mild suicidal feelings than when they did not have suicidal feelings (OR = 1.19, 95% CI: 1.02–1.39, *p*<0.05). However, a striking finding was that the rate was significantly reduced in pure victims among the adolescents when they had suicidal feelings compared with when they did not have suicidal feelings (OR = 0.47, 95% CI: 0.23–0.94, *p*<0.05 for mild suicidal feelings, and OR = 0.32, 95% CI: 0.16–0.67, *p* = 0.002 for serious suicidal feelings, respectively). This effect of suicidal feelings in the bullied adolescents was due to the fact that there was no increase in help-seeking, which was higher in the bullied adolescents without suicidal feelings, as summarized in Table 3. As shown in Table 2, the pure victims mostly sought informal help (144 of the 261 pure victims) when they sought any help. Among the informal help-seeking, the most frequent source of help was peers (n = 115), followed by family members (n = 66), while a few sought help from school teachers (n = 15). The number of pure victims who sought formal help was also low (29 out of 261). As summarized in Figure 2, seeking help from peers and family members was significantly reduced in the victims with serious suicidal feelings (OR = 0.21, 95% CI: 0.09–0.51, *p*<0.01 and OR = 0.13, 95% CI: 0.04–0.45, *p*<0.001, respectively), compared to those without suicidal feelings.

**Table 3:** The rate of middle(-late) adolescents who sought help for psychological distress, according to bullying status and suicidal ideation.

	Total of subjects		Uninvolved		Pure bullies		Pure victims		Bully-victims	
Total	3161/8407	(37.6)	2844/7734	(36.8)	131/318	(41.2)	142/261	(54.4)	44/94	(46.8)
Suicidal feelings										
None^a^	2701/7300	(37.0)	2470/6823	(36.2)	99/245	(40.4)	104/170	(61.2)^b^	28/62	(45.2)
Mild	314/725	(43.3)	266/629	(42.3)	17/35	(48.6)	22/47	(46.8)	9/14	(64.3)
Serious	146/382	(38.2)	108/282	(38.3)	15/38	(39.5)	16/44	(36.4)	7/18	(38.9)

*Note*. The number of subjects who sought help, divided by the total number of adolescents, is shown by bullying status and suicidal feelings, with the rate (%) in brackets. Without suicidal feelings, the rate of the subjects who sought help was significantly associated with bullying status in middle adolescents (^a^ *p*<0.001, Kruskal-Wallis test), while the rate in pure victims was significantly higher than the uninvolved and pure bullies (^b^ *p*<0.001, Bonferroni post hoc test). With suicidal feelings, however, the rate did not increase among victims of bullying. (The total number of subjects is less than the number of subjects analyzed due to missing data for suicidal feelings and bullying status.)

## Discussion

The present study first investigated the effects of bullying status and suicidal feelings on actual help-seeking behavior in middle(-late) adolescents (aged 15–18 years). Cross-sectional data from a large school-based sample were analyzed. The rate of adolescents who actually sought help was significantly higher in the bullied adolescents compared with the uninvolved and bullying adolescents. What was more striking, however, was that the higher level of help-seeking behavior in the bullied (or pure victims) disappeared when they had suicidal feelings. This interaction between being bullied and having suicidal feelings was more noticeable when the suicidal feelings were serious, rather than mild. The effect was more noticeable when suicidal feelings were serious. Hence, the present study suggests that in bullied middle(-late) adolescents, having suicidal feelings may obstruct the increase in help-seeking behavior. The present finding may have a significant impact on the prevention of suicidal behaviors, because middle-late adolescence is an age when the risk of suicide sharply increases[2], [35] and help-seeking behavior may be of great significance in the prevention of suicide at this age. In addition, suicidal feelings were more frequent in the victims of bullying than in the uninvolved and in bullies, consistent with previous studies[10], [17]–[21]. To the best of our knowledge, the present study is the first to observe the change in help-seeking in bullied adolescents with suicidal feelings.

Except for the middle(-late) adolescents with suicidal feelings, help-seeking behavior was higher in bullied adolescents. The relation between bullying and help-seeking has not been clarified. Several studies have reported that bullied children and adolescents may hesitate to consult others [25], [26]. Other studies, however, have observed that they may seek more help when bullied [28]–[30]. The present results are consistent with the latter studies, but further research is required to draw a definite conclusion.

Regarding the relation between suicidal feelings and help-seeking behavior, the rate of adolescents who sought help was higher when they had mild suicidal feelings. This elevation was not observed when suicidal feelings were serious. A previous epidemiological study found that significantly more adolescents with suicidal feelings sought help than those without such feelings [31]. Studies investigating the intention to seek help, not actual behavior, however, have made contrasting findings [32], [33], [35], [36]. The present results are partly consistent with the previous studies that investigated actual behavior. A higher rate of help-seeking behavior was, however, not observed in the adolescents with serious suicidal feelings in the present study.

The most frequent sources of help in the bullied middle(-late) adolescents were peers and family members. The help-seeking behavior directed toward these informal sources was significantly reduced when the bullied middle(-late) adolescents had suicidal feelings. In contrast, formal help-seeking (e.g., from school nurses and health care professionals) was rare among the pure victims, regardless of the presence or absence of suicidal feelings. The decrease in help-seeking (mostly informal help-seeking) in those with suicidal feelings may be related to the previous observations of elevated isolation in adolescents with suicidal feelings [37], [38]. Isolation is a major factor in committing suicide [5], [39], [40]. The decreased help-seeking from peers and family members in those with suicidal feelings may enhance the isolation at school and at home, which could block the channel of seeking help at the time of great risk of committing suicide.

The following limitations must be acknowledged. First, this is a cross-sectional survey, and therefore the causal relationships among suicidal feelings, bullying and help-seeking are not clear. Second, the participants were asked about help-seeking for “psychological stress or problems” in the questionnaire. Help-seeking for suicidal feelings or bullying was not asked about directly. This could affect the observed associations between suicidal feelings, bullying and help-seeking. Third, we did not ask the participants about the detailed features of bullying, including frequency, duration or type (e.g., physical, verbal, relational, or cyber bullying). Bullying-type specific relations between suicidal feelings and help-seeking remain to be further studied. Fourth, we used a simple sentence to ask about suicidal feelings. Therefore, we were not able in the present study to discriminate between non-imminent suicidal feelings and imminent severe suicidal ideas which lead to real suicide attempts. The effect of having such imminent, suicidal ideas should be further studied. Fifth, the present findings could be specific to the Japanese middle(-late) adolescents.

The present study suggests that in bullied middle(-late) adolescents, having serious suicidal feelings may not increase help-seeking behaviors. This finding is critically important, because this means that help-seeking behavior may not be increased in the adolescents in the most need of the help. Health care professionals and school staff should be aware of this finding and may need to have appropriate skills to inquire about suicidal feelings or ideas in adolescents, especially in those being bullied.

## References

[1] WHO Health statistics and health information system [mortality database].

[2] Patton G, Coffey C, Sawyer S, Viner R, Haller D, et al (2009) Global patterns of mortality in young people: a systematic analysis of population health data. Lancet 374: 881–892. 19748397 10.1016/S0140-6736(09)60741-8

[3] Hawton K, Heeringen K (2009) Suicide. Lancet 373: 1372–1381. 19376453 10.1016/S0140-6736(09)60372-X

[4] Japan Ministry of Health Labour and Welfare (2011) Vital Statistics.

[5] WHO (2012) Public health action for the prevention of suicide a framework.

[6] Hawton K, Saunders K, O'Connor R (2012) Self-harm and suicide in adolescents. Lancet 379: 2373–2382. 22726518 10.1016/S0140-6736(12)60322-5

[7] Qin P, Argerbo E, Mortensen P (2005) Factors contributing to suicide: the epidemiological evidence from large-scale registers; K H, editor. New York: Oxford University Press. 2–28 p.

[8] Arseneault L, Bowes L, Shakoor S (2010) Bullying victimization in youths and mental health problems: ‘much ado about nothing’? Psychological Medicine 40: 717–729. 19785920 10.1017/S0033291709991383

[9] Michelmore L, Hindley P (2012) Help-seeking for suicidal thoughts and self-harm in young people: a systematic review. Suicide and Life Threatening Behavior 42: 507–524. 22889130 10.1111/j.1943-278X.2012.00108.x

[10] Hepburn L, Azrael D, Molnar B, Miller M (2012) Bullying and suicidal behaviors among urban high school youth. Journal of Adolescent Health 51: 93–95. 22727083 10.1016/j.jadohealth.2011.12.014

[11] Gulliver A, Griffiths K, Christensen H, Brewer J (2012) A systematic review of help-seeking interventions for depression, anxiety and general psychological distress. BMC Psychiatry 12: 81. 22799879 10.1186/1471-244X-12-81 PMC3464688

[12] Rickwood D, Frank D, Wilson C (2007) When and how do young people seek professional help for mental health problems? Medical Journal of Australia 187. 10.5694/j.1326-5377.2007.tb01334.x 17908023

[13] Mann J, Apter A, Bertolote J, Beautrais A, Currier D, et al (2005) Suicide Prevention Strategies A Systematic Review. Journal of the American Medical Association 294: 2064. 16249421 10.1001/jama.294.16.2064

[14] Rickwood D, Deane F, Wilson C, Ciarrochi J (2005) Young people's help-seeking for mental health problems. Australian e-Journal for the Advancement of Mental Health 4.

[15] Marcel F. van der Wal, Cees A. M. de Wit, Remy A Hirasing (2003) Psychosocial Health Among Young Victims and Offenders of Direct and Indirect Bullying. Pediatrics 111: 1312–1317. 12777546 10.1542/peds.111.6.1312

[16] Craig W, Harel-Fisch Y, Fogel-Grinvald H, Dostaler S, Hetland J, et al (2009) A cross-national profile of bullying and victimization among adolescents in 40 countries. International Journal of Public Health 54 Suppl 2216–224. 19623475 10.1007/s00038-009-5413-9 PMC2747624

[17] Kim Y, Koh Y, Leventhal B (2005) School bullying and suicidal risk in Korean middle school students. Pediatrics 115: 357–363. 15687445 10.1542/peds.2004-0902

[18] Kaltiala-Heino R, Rimpela M, Marttunen M, Rimpela A, Rantanen P (1999) Bullying, depression, and suicidal ideation in Finnish adolescents: school survey. British Medical Journal 319: 348–351. 10435954 10.1136/bmj.319.7206.348 PMC28187

[19] Klomek A, Marrocco F, Kleinman M, Schonfeld I, Gould M (2007) Bullying, depression, and suicidality in adolescents. Journal of the American Academy of Child & Adolescent Psychiatry 46: 40–49. 17195728 10.1097/01.chi.0000242237.84925.18

[20] Klomek A, Marrocco F, Kleinman M, Schonfeld I, Gould M (2008) Peer Victimization, Depression, and Suicidiality in Adolescents. Suicide and Life-Threatening Behavior 38. 10.1521/suli.2008.38.2.166 18444775

[21] Klomek A, Sourander A, Niemela S, Kumpulainen K, Piha J, et al (2009) Childhood bullying behaviors as a risk for suicide attempts and completed suicides: a population-based birth cohort study. American Academy of Child & Adolescent Psychiatry 48: 254–261. 10.1097/CHI.0b013e318196b91f 19169159

[22] Fisher H, Moffitt T, Houts R, Belsky D, Arseneault L, et al (2012) Bullying victimisation and risk of self harm in early adolescence: longitudinal cohort study. British Medical Journal 344: e2683–e2683. 22539176 10.1136/bmj.e2683 PMC3339878

[23] Arseneault L, Walsh E, Trzesniewski K, Newcombe R, Caspi A, et al (2006) Bullying victimization uniquely contributes to adjustment problems in young children: a nationally representative cohort study. Pediatrics 118: 130–138. 16818558 10.1542/peds.2005-2388

[24] Heikkila HK, Vaananen J, Helminen M, Frojd S, Marttunen M, et al (2013) Involvement in bullying and suicidal ideation in middle adolescence: a 2-year follow-up study. Eur Child Adolesc Psychiatry 22: 95–102. 23053774 10.1007/s00787-012-0327-0

[25] Oliver C, Candappa M (2007) Bullying and the politics of ‘telling’. Oxford Review of Education 33: 71–86.

[26] Boulton M, Underwood K (1992) Bully/Victim Problems Among Middle School Children. British Journal of Psychology. 10.1111/j.2044-8279.1992.tb01000.x 1558813

[27] Smith P, Shu S (2000) What Good School can Do About Bullying Findings from a Survey in English Schools After a Decade of Research and Action. Childhood 7: 193–212.

[28] Hunter S, Boyle J, Warden D (2004) Help seeking amongst child and adolescent victims of peer-aggression and bullying: The influence of school-stage, gender, victimisation, appraisal, and emotion. British Journal of Educational Psychology 74: 375–390. 15296546 10.1348/0007099041552378

[29] Haavet O, Straand J, Hjortdahl P, Saugstad O (2005) Do negative life experiences predict the health-care-seeking of adolescents? A study of 10th-year students in Oslo, Norway. Journal of Adolescent Health 37: 128–134. 16026722 10.1016/j.jadohealth.2004.08.031

[30] Eliot M, Cornell D, Gregory A, Fan X (2010) Supportive school climate and student willingness to seek help for bullying and threats of violence. Journal of School Psychology 48: 533–553. 21094397 10.1016/j.jsp.2010.07.001

[31] Husky M, Olfson M, He J, Nock M, Swanson S, et al (2012) Twelve-Month Suicidal Symptoms and Use of Services Among Adolescents: Results From the National Comorbidity Survey. Psychiatric Services. 10.1176/appi.ps.201200058 PMC5100004 22910768

[32] Wilson C, Deane F, Marshall K, Dalley A (2010) Adolescents' suicidal thinking and reluctance to consult general medical practitioners. Journal of Youth and Adolescence 39: 343–356. 20229227 10.1007/s10964-009-9436-6

[33] Gould M, Velting D, Kleinman M, Lucas C, Thomas JG, et al (2004) Teenagers' attitudes about coping strategies and help-seeking behavior for suicidality. Journal of the American Academy of Child & Adolescent Psychiatry 43: 1124–1133. 15322416 10.1097/01.chi.0000132811.06547.31

[34] Paykel ES, Myers JK, Lindenthal JJ, Tanner J (1974) Suicidal Feelings in the General Population: A Prevalence Study. The British Journal of Psychiatry 124: 460–469. 4836376 10.1192/bjp.124.5.460

[35] Wilson C, Deane F, Ciarrochi J (2005) Can hopelessness and adolescents' beliefs and attitudes about seeking help account for help negation? Journal of Clinical Psychology 61: 1525–1539. 16173086 10.1002/jclp.20206

[36] Deane F, Wilson C, Ciarrochi J (2001) Suicidal Ideation and Help-Negation: Not Just Hopelessness or Prior Help. Journal of Clinical Psychology 57: 901–914. 11406803 10.1002/jclp.1058

[37] Pompili M, Forte A, Palermo M, Stefani H, Lamis D, et al (2012) Suicide risk in multiple sclerosis: a systematic review of current literature. Journal of Psychosomatic Research 73: 411–417. 23148807 10.1016/j.jpsychores.2012.09.011

[38] Johnson J, Cohen P, Gould M, Kasen S, Brown J, et al (2002) Suicide and psychiatric diagnosis: a worldwide perspective. Archives of General Psychiatry 59: 741–749. 12150651 10.1001/archpsyc.59.8.741

[39] Cash S, Bridge J (2009) Epidemiology of youth suicide and suicidal behavior. Current Opinion in Pediatrics 21: 613–619. 19644372 10.1097/MOP.0b013e32833063e1 PMC2885157

[40] King M, Semlyen J, Tai S, Killaspy H, Osborn D, et al (2008) A systematic review of mental disorder, suicide, and deliberate self harm in lesbian, gay and bisexual people. BMC Psychiatry 8: 70. 18706118 10.1186/1471-244X-8-70 PMC2533652

