# Correction: Suicidal Feelings Interferes with Help-Seeking in Bullied Adolescents

**DOI:** 10.1371/journal.pone.0113187

**Published:** 2014-11-05

**Authors:** The PLOS ONE Staff

There is an error in the title. The correct title is: Suicidal Feelings Interfere with Help-Seeking in Bullied Adolescents.

The correct citation is: Kitagawa Y, Shimodera S, Togo F, Okazaki Y, Nishida A, et al. (2014) Suicidal Feelings Interfere with Help-Seeking in Bullied Adolescents. PLoS ONE 9(9): e106031. doi:10.1371/journal.pone.0106031.

Additionally, one of the affiliations for the corresponding author is missing. Yuko Kitagawa is affiliated with both 1. Department of Health Education, Graduate School of Education, University of Tokyo, Tokyo, Japan and 2. Research Fellow of Japan Society for the Promotion of Science.
